# Permeable Hydrogel Encapsulated Osteosarcoma‐on‐a‐Chip for High‐Throughput Multi‐Drugs Screening

**DOI:** 10.1002/smmd.70013

**Published:** 2025-07-12

**Authors:** Haiwen Su, Yuanhai Chen, Zhiyan Xuan, Haoyu Ren, Peihua Lu, Miaoqing Zhao, Huan Wang

**Affiliations:** ^1^ The Eighth Affiliated Hospital Sun Yat‐sen University Shenzhen China; ^2^ Affiliated Hospital of Guangdong Medical University Guangdong Medical University Zhanjiang China; ^3^ Department of Oncology Wuxi Medical Center The Affiliated Wuxi People’s Hospital of Nanjing Medical University Wuxi People’s Hospital Nanjing Medical University Wuxi China; ^4^ Department of Pathology Shandong Cancer Hospital and Institute Shandong First Medical University and Shandong Academy of Medical Sciences Jinan China

**Keywords:** electrospray, microcarriers, microfluidics, organ‐on‐a‐chip, osteosarcoma

## Abstract

Pharmacological chemotherapy remains a cornerstone in treating osteosarcoma (OS), where the application of drug combinations not only enhances therapeutic efficacy but also mitigates adverse side effects. However, the absence of an efficient and reliable drug screening platform poses a significant challenge in optimizing these combination therapies. In this study, we introduce a novel OS chip designed to facilitate the high‐throughput and precise evaluation of drug combination efficacy, addressing this critical gap in OS treatment research. Leveraging the precise control of microfluidic electrospray technology, we successfully fabricated core–shell microcarriers (CSMs) encapsulating OS cells with uniform size and monodisperse distribution. Through three‐dimensional culture, OS cell spheroids were efficiently formed within the CSMs, which were subsequently integrated into a microfluidic chip equipped with a concentration gradient generator and cell culture chambers. This innovative platform enables high‐throughput screening of single drugs and diverse drug combinations. Our results demonstrate that this OS‐on‐a‐chip exhibits significant potential for clinical drug screening, offering a robust tool for optimizing therapeutic strategies in OS treatment.

## Summary

- Innovative osteosarcoma‐on‐a‐chip for high‐throughput drug screening
- Core–shell microcarriers enable uniform 3D OS spheroid formation
- Microfluidic electrospray technology for precise drug combination testing
- Concentration gradient generator enhances multi‐drug efficacy evaluation

## Graphical abstract

This study presents an osteosarcoma (OS) chip for high‐throughput screening of drug combinations, combining microfluidic electrospray technology with core–shell microcarriers (CSMs) to form uniform OS spheroids. The chip integrates gradient generator and culture chambers, enabling precise evaluation of single and combination drug efficacy. This platform offers a clinically relevant tool to optimize OS chemotherapy with enhanced efficiency and reliability.

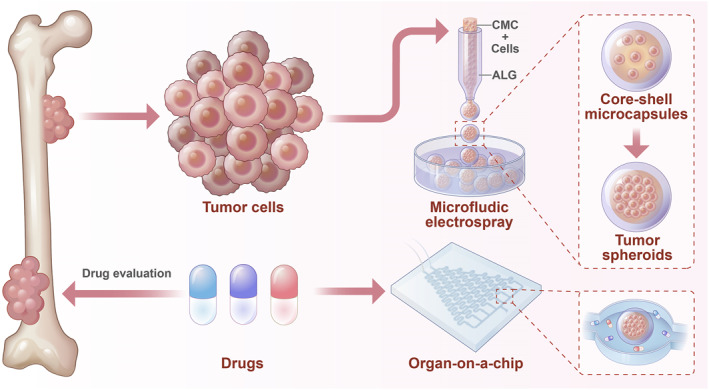

## 1 Introduction

Osteosarcoma (OS) is one of the most common malignant bone tumors and it prevails in highly active adolescents or children under 20 years of age [1, 2, 3, 4, 5]. It characteristically originates from within the bone, exhibiting rapid growth, high aggressiveness, and a high tendency to metastasize [6, 7, 8], which contributes to significant physical and psychological distress for the affected individuals and imposes considerable financial burden on healthcare systems. Despite advances in medical technology, the prognosis for OS remains far from optimistic, with recurrence being a frequent occurrence [9, 10, 11, 12], and some patients even exhibit chemoresistance [13, 14, 15, 16]. Therefore, it is urgent to improve the treatment strategies for OS. Recent advances in microfluidic technologies have opened new possibilities for creating more physiologically relevant tumor models that better mimic the complex in vivo microenvironment. The development of in vitro drug evaluation platforms has shown great significance in guiding clinical drug usage. Recently, some preclinical models have been reported for in vitro drug evaluation, but they are limited by their reliance on two‐dimensional (2D) cell cultures or animal models [17, 18, 19, 20, 21, 22, 23, 24, 25], which are unable to replicate the dynamic tumor microenvironment and spatial interactions between cells [26, 27, 28, 29, 30, 31, 32, 33]. Additionally, species differences in animal models often hinder the translation of drug development findings [34, 35, 36]. These limitations highlight the critical need for innovative platforms that can bridge the gap between traditional models and clinical applications. As a result, there is an urgent need to develop effective drug evaluation platforms for studying OS treatment strategies.

**FIGURE 1:**
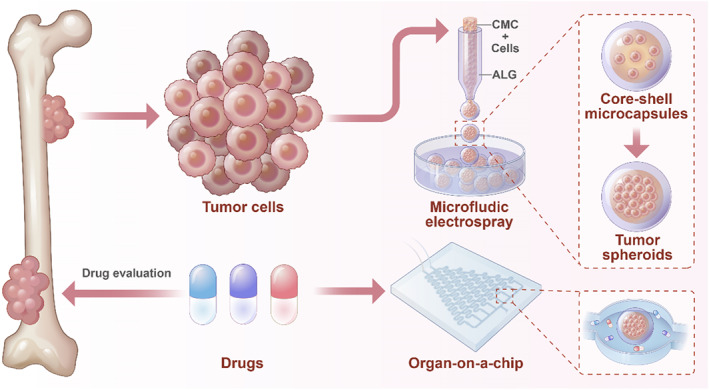
Schematic diagram of the OS‐on‐a‐chip preparation process and drug evaluation.

We present an advanced platform combining microfluidics and hydrogel encapsulation technologies to generate organ‐specific (OS) three‐dimensional (3D) tumor models, offering transformative potential for conventional drug evaluation paradigms (Figure 1). Particularly, 3D models, such as tumor spheroids, have been shown to more closely resemble the in vivo architecture of tumors and to be more biologically relevant than 2D cell cultures, which do not have the interactions of the real tumor microenvironment [37, 38, 39, 40]. 3D spheroids allow for dynamic cell interactions with the extracellular matrix‐driving cellular activities of growth, migration, and invasion, which are quintessential for understanding tumor progression. Besides, these models can replicate the heterogeneity of cancer, and thus, they have become a pre‐requisite tool in drug discovery. However, to date, most 3D tumor spheroids have been cultured in simple systems with minimal structural confinement and spatial organization, hindering precise control of nutrient and drug distribution across the model [41, 42, 43]. In addition, the natural variability exhibited in the sizes and cell types of spheroids further limits their predictive capability related to individual patient tumors. In contrast, microfluidic technology is promising for offering tight control over fluidic conditions in geometries at the microscale level [44, 45, 46, 47, 48, 49, 50]. This enables optimized dynamic delivery of nutrients and drugs, accurately replicating human physiological conditions. Additionally, microfluidics facilitates the generation of uniform and reproducible microcapsules with customized structural properties, thereby improving the fidelity of 3D tumor models [51, 52, 53, 54, 55]. The integration of these technologies therefore endows our platform with a highly controlled and reproducible system for OS drug evaluation, improving the accuracy and predictive power of preclinical screening.

In this work, we present a novel approach for preparing core–shell microcarriers (CSMs) containing OS cells for drug evaluation. It was realized by using the microfluidic electrospray method to encapsulate human OS cells into CSMs, achieving sodium alginate (ALG) as the outer phase and carboxymethyl cellulose (CMC) combined with OS cells as the inner phase. This approach enabled the production of CSMs with consistent size and morphology, offering a robust platform for 3D cell culture. Encapsulated OS cells could proliferate, form spheroids, and replicate critical features of OS tumors. We then introduced these CSMs into a dynamic drug screening system, providing a high‐throughput reproducible method for testing anti‐tumor agents. This development not only facilitates the creation of more accurate preclinical models of OS but also represents a step toward improving the accuracy and safety of drug evaluation in cancer treatment.

## 2 Methods

### 2.1 Materials

Sodium alginate (ALG), carboxymethyl cellulose (CMC), calcium chloride (CaCl_2_), ethylene diamine tetraacetic acid (EDTA) were purchased from Shanghai Aladdin Reagent Co. MEM medium, fetal bovine serum, penicillin‐streptomycin mixture, sodium bicarbonate, and sodium pyruvate were purchased from Gibco Life Technologies, USA. Live/dead kit and CCK‐8 assay kit were purchased from Shanghai Beyotime Biotechnology Co. Cisplatin, doxorubicin and ifosfamide were purchased from Shanghai BiDe Pharmaceutical Technology Co. CoraLite Plus 488‐phalloidin sourced from Wuhan Sanying Company, China. Triton X‐100 sourced from Solarbio, China.

### 2.2 Fabrication of Core–Shell Microcarriers (CSMs)

In typical experiments, CSMs were prepared by microfluidic electrospray technology. First, an inner tube (200 μm) was nested in an outer tube (500 μm) to form a microfluidic chip. Next, a voltage was applied between the microfluidic chip outlet and the collection phase (2% CaCl_2_ solution) to form an electric field. Then, 1.5% ALG solution and 1% CMC solution were used as the outer phase and the inner phase, respectively, at a controlled flow rate ratio. The outer phase solution wrapped around the inner phase solution, and the core–shell fluid was separated into core–shell droplets under the action of an electric field. Finally, the core–shell microcarriers were formed in the collection phase by the fast crosslinking caused by Ca^2+^.

### 2.3 Characterizations of Core–Shell Microcarriers

The surface and internal morphology of the core–shell microcarriers were characterized by scanning electron microscope (SEM). The mechanical properties of the ALG microcarriers were evaluated using a universal testing machine. The ALG samples had a diameter of 5 mm, a height of 2 mm and a compression ratio of 1 mm/min.

### 2.4 hOS Cell Spheroids Culture

The cell spheroids were mainly prepared using the core–shell microcarrier preparation method described above. Equal volumes of 2% CMC and culture medium with an appropriate density of hOS cells were mixed thoroughly. The resulting solution was used as the inner phase for the microfluidic device. After collecting the core–shell microcarriers containing hOS cells from the collection phase, they were transferred to cell culture dishes. The microcarriers were then rinsed several times with fresh culture medium and cultured at 37°C with 5% CO_2_. The cell culture medium was MEM medium containing 10% FBS, 1% penicillin‐streptomycin mixture (100X), 2% sodium bicarbonate, and 1% sodium pyruvate.

### 2.5 hOS Cell Spheroids Viability

The core–shell microcarriers containing cell spheroids were transferred from the medium and placed on a cell crawler, and incubated for 30 min away from light using a live/dead kit. Finally, the cells were observed under a confocal microscope, in which green fluorescence was for live cells and red fluorescence was for dead cells. To assess the viability of hOS cell spheroids, the core–shell microcarriers were initially immersed in a 2% EDTA solution to remove the hydrogel shell layer. This process facilitated the release of the cell spheroids. The viability of the resulting cell spheroids was then evaluated using the CCK‐8 Assay Kit.

### 2.6 Immunofluorescence Staining of Cytoskeleton

The hOS cell spheroids were washed three times with PBS to remove residual media. Subsequently, the spheroids were fixed on ice using a PBS solution containing 4% paraformaldehyde for 15 min, followed by another three washes with PBS. To permeabilize the spheroids, they were treated with PBS containing 0.2% Triton X‐100 for 5 min at room temperature and then washed again with PBS three times. Next, 5 μL of fluorescence‐labeled phalloidin stock solution was diluted in 200 μL of PBS, added to one coverslip, and incubated for 20 min at room temperature for staining. After that, the spheroids were washed 3 times with PBS. Finally, fluorescence microscopy was performed to observe the stained hOS cell spheroids.

### 2.7 Design of Microfluidics‐Based Organ‐on‐a‐Chip

The microfluidic chip consisted of two inflow channels, one outflow channel, a concentration generator, and eight cylindrical cell culture chambers. The inflow channels were designed to selectively introduce various drugs or cell culture medium. These inputs were then processed through a concentration gradient generator, which established appropriate cell culture conditions or created a drug concentration gradient.

### 2.8 Evaluation of Drug Efficacy

Cell spheroids were cultured within the cylindrical chambers of the microfluidic chip. One inflow channel was supplied with various drugs, while the other was fed with cell culture medium. Concentration gradient generator facilitated the formation of different drug concentrations. The chip was then incubated at 37°C with 5% CO_2_. After the designated treatment period, drug efficacy was evaluated using a live/dead assay kit.

## 3 Result and Discussion

**FIGURE 2:**
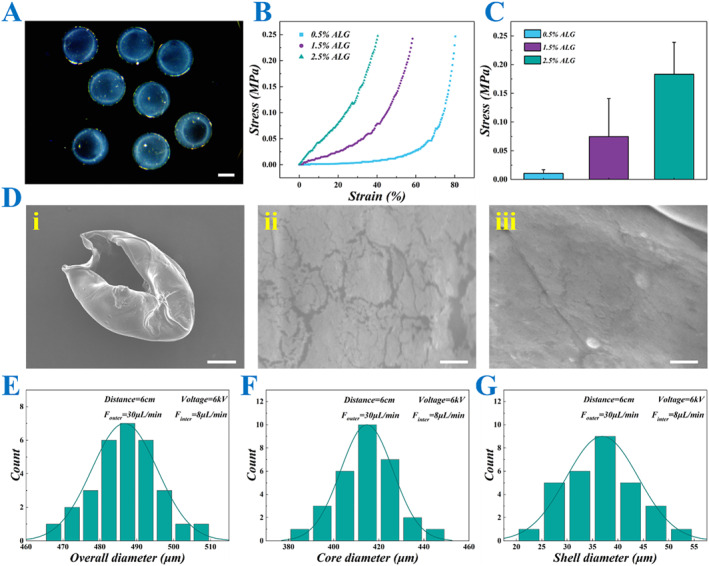
Characterization of CSMs. (A) The image of CSMs under stereomicroscope. (B) The stress‐strain curves of the hydrogels with different concentrations of ALG. (C) The quantitative data on the stress of ALG hydrogels with different concentrations. (D) The SEM images of the CSMs. (i) The overall appearance, (ii) the outer surface appearance, (iii) the inner surface appearance. (E–G) The overall (E), core (F) and shell (G) size distributions of the CSMs prepared under specific parameter conditions. Scale bars represent 200 μm in (A), 100 μm in (D, i) and 2 μm in (D, ii and iii).

In a typical experiment, microfluidics combined with electrospray technology was used to prepare hydrogel microcarriers with a core–shell hierarchical structure. First, a microfluidic chip was designed with nested inner and outer tubes. The outer tube and the inner tube were filled with ALG solution and CMC solution, respectively, and both of them were injected using a micro syringe pump, while the collection phase was CaCl_2_ solution. To induce the rapid crosslinking, an electric field was applied between the collection phase and the outlet of the microfluidic chip. The electric field cut the fluid at the end of the chip quickly, and ejected the generated droplets into the collection phase, where they underwent rapid crosslinking to form CSMs (Figure 2A). To confirm the core–shell structure of the CSMs, scanning electron microscope (SEM) was employed for characterization, which clearly revealed the distinct separation between the core and shell, thus further validating the successful fabrication of the microcarriers (Figure 2D). To fabricate CSMs with an optimal size and core‐to‐shell ratio, we systematically optimized the inner and outer flow rates and applied voltage. The corresponding results are presented in Supporting Information S1: Figure S1A,B. It was observed that increasing the inner flow rate led to a gradual increase in the core diameter, while the total diameter of the microcarriers showed neglectable changes. Similarly, increasing the outer flow rate led to a significant increase in the overall diameter of the microcarriers (Supporting Information S1: Figure S1C). Additionally, an increase in voltage caused a larger electric field force, which subsequently reduced the droplet size (Supporting Information S1: Figure S1D). Besides, the CSMs had a uniform size, ensuring consistency in their application for cell encapsulation and drug evaluation (Figure 2E–G). Overall, by adjusting the experimental conditions, the size and core‐to‐shell ratio of the CSMs can be tailored to suit specific application needs.

Next, an investigation was conducted into the mechanical strength of the hydrogel of CSMs with varying concentrations of ALG. The results presented in Figure 2B,C reveal that the stress response increased with the concentration of ALG as observed in the classical stress‐strain curves. Hydrogels with higher ALG concentrations exhibit greater stress at similar strain levels, indicating enhanced mechanical strength and stiffness. Among the three tested concentrations, the 2.5% ALG hydrogel demonstrated the highest stress values. However, the primary goal was to achieve appropriate mechanical strength while maintaining optimal maneuverability and biocompatibility. Although higher concentrations of ALG provide superior mechanical strength, they result in excessive rigidity, which may impede the adaptability of the cell culture environment and the stability of the microenvironment. Additionally, higher ALG concentrations can complicate the cross‐linking process and reduce the plasticity of the hydrogels during microcapsule preparation. Therefore, in the subsequent experiments, we selected a 1.5% ALG concentration, as it offered a better balance between mechanical properties and biological functions.

**FIGURE 3:**
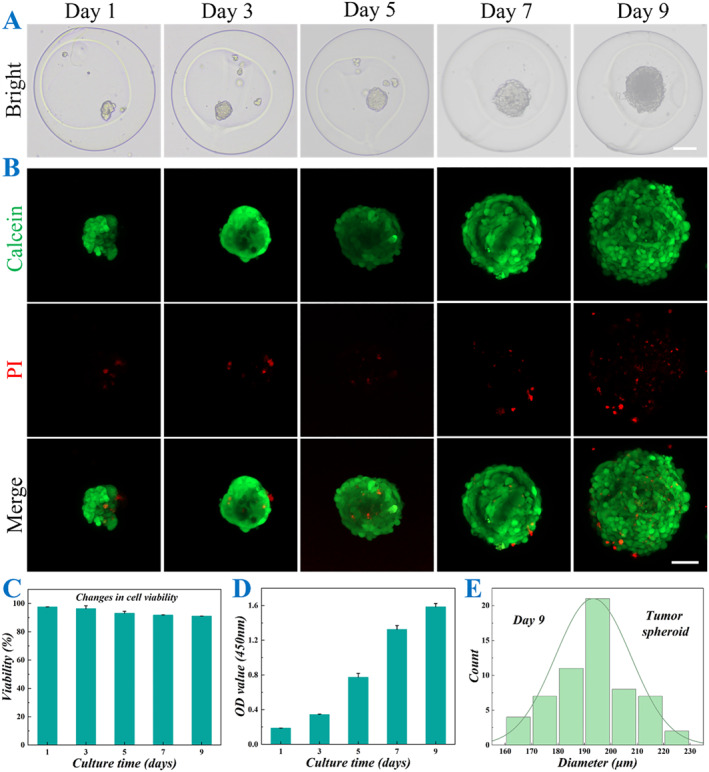
Culture and characterization of hOS cell spheroids in CSMs. (A) Bright field images of CSMs loaded with hOS cell spheroids after 1, 3, 5, 7, and 9 days of culture. (B) Fluorescent images of live‐dead staining of CSMs containing hOS cell spheroids after 1, 3, 5, 7, and 9 days of culture. (C) Quantitative analysis of live hOS cell spheroids on days 1, 3, 5, 7, and 9 (*n* = 3 for each group). (D) Cell viability of hOS cell spheroids by CCK‐8 assay on days 1, 3, 5, 7, and 9 (*n* = 3 for each group). (E) Diameter distribution of hOS cell spheroids on day 9. Scale bars represent 100 μm in (A) and 50 μm in (B).

By using the properly mixed hOS cell suspension and CMC solution as the inner fluid in the microfluidic electrospray system, the hOS cells were successfully encapsulated into the core of CSMs. To evaluate the biocompatibility and 3D culture conditions of the microcarriers, the CSMs containing hOS cells were cultured for 9 days. During this period, 3D cell spheroids began to form and gradually enlarge, as shown in Figure 3A. Furthermore, live‐dead staining was performed on the encapsulated cell spheroids, and the results confirmed that the cell spheroids maintained good viability, indicating the good effectiveness of the CSMs in supporting cell growth and functions in the 3D culture environment (Figure 3B–D). Additionally, the sizes of cell spheroids were monitored from day 1 to day 9. The diameters increased from over 50 μm on the first day of culture to more than 200 μm by day 9 (Supporting Information S1: Figure S2). Furthermore, diameter distribution analysis on day 9 revealed a relatively narrow size range, with the majority of measurements clustered around 200 μm. The consistent size distribution suggests a high degree of uniformity in the cellular aggregation process, which is crucial for ensuring experimental reproducibility and reliability in subsequent functional analyses (Figure 3E).

Since microfluidic electrospray technology utilizes a high‐voltage power supply during the preparation of cell‐loaded CSMs, it is crucial to address potential concerns regarding its impact on cell viability. For this purpose, cell‐loaded CSMs were prepared under varying voltage and cultured in vitro for several days, and live‐dead staining was performed to assess the proportion of viable cells within the spheroids. As shown in Supporting Information S1: Figure S3, the viability of cells in all voltage groups exceeded 90%, demonstrating that the voltage had no significant adverse effect on cell spheroid growth.

**FIGURE 4:**
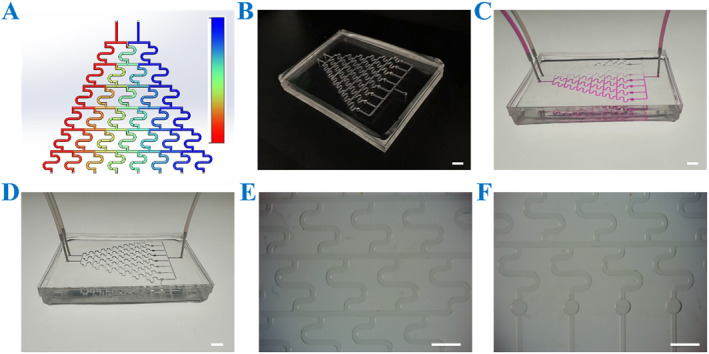
Characterization of the microfluidic chip. (A) The numerical simulation of the concentration gradient generator, with the color gradient (blue to red) representing the increasing solute concentration. (B) The overall appearance of the chip. (C) The overall image of the chip passed through the rhodamine solution. (D) The image of the chip connecting the input and output channels. (E) The image of the concentration generator under a stereomicroscope. (F) The image of a cell culture chamber under a stereomicroscope. Scale bars are 5 mm in (B–D) and 3 mm in (E and F).

To better simulate the in vivo drug delivery process and enhance the physiological relevance of drug evaluation, a novel microfluidic chip was tailored to integrate with the CSMs to form an OS‐on‐a‐chip. This advanced chip integrates two core parts: a concentration gradient formation system and a cell culture system. The concentration gradient formation system leverages hydrodynamic flow focusing to generate precise and reproducible drug concentration gradients, while the cell culture system facilitates the simulation of spatiotemporal cellular interactions and the recreation of extracellular microenvironments. Together, these features provide a robust and physiologically relevant platform for comprehensive drug assessment. As illustrated in Figure 4, the microfluidic platform comprises eight parallel microchannels that establish discrete and stable concentration gradients through hydrodynamic flow focusing. Each gradient channel is seamlessly interfaced downstream with the corresponding cell culture chambers, ensuring precise delivery of drug concentrations to the target cells. This innovative architecture not only enables the simultaneous generation of eight independent drug concentration profiles but also maintains spatial isolation among test conditions, thereby minimizing cross‐contamination and enhancing experimental reproducibility. By closely mimicking the dynamic and complex nature of in vivo drug delivery, this platform offers a significant advancement in preclinical drug evaluation, bridging the gap between traditional in vitro assays and in vivo studies.

**FIGURE 5:**
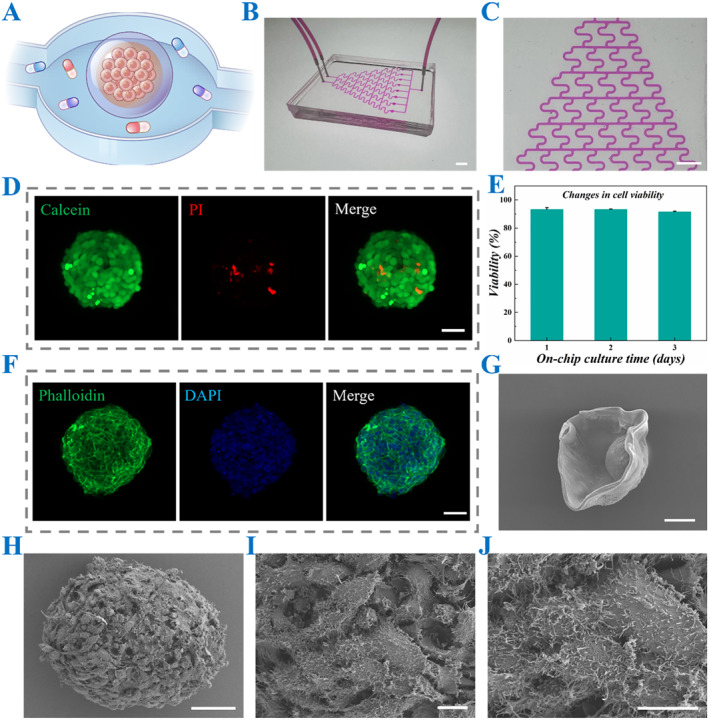
Characterization of CSMs loaded with cell spheroids cultured in chips. (A) The schematic diagram of CSMs loaded with cell spheroids inside a cell culture chamber. (B and C) The images of rhodamine‐imitating cell cultures filling the channel (B) as well as the images under the stereomicroscope (C). (D) Live‐dead diagram of cell spheroids after incubation in a cell culture chamber. (E) Quantification of live‐dead cell spheroids cultured for 3 days in cell culture chambers (*n* = 3 for each group). (F) The immunofluorescence image of the cytoskeleton. (G) The SEM image of a CSM loaded with a cell spheroid. (H–J) The images of a cell spheroid under a SEM, with localized magnified details. Scale bars are 5 mm in (B and C), 50 μm in (D, F, H), 100 μm in (G) and 10 μm in (I and J).

Typically, the cell spheroid‐loaded CSMs were cultured within the designated cell culture chambers of the microfluidic chip (Figure 5A). Subsequently, the chip was interfaced with external perfusion systems, and cell culture medium was introduced through the inlet ports, establishing dynamic culture conditions with controlled flow rates (Figure 5B,C). To assess the viability of cell spheroids cultured under dynamic conditions, live/dead staining was conducted following 3 days of continuous perfusion (Figure 5D,E). The homogeneous distribution of viable cells (green fluorescence) throughout the spheroid architecture demonstrated efficient nutrient transport and metabolic waste removal mediated by the perfusion system. Cytoskeletal staining results indicated that the cultured spheroids had well‐preserved structural organization with distinct spatial patterns of cytoskeletal components (Figure 5F and Supporting Information S1: S4). These observations confirmed the maintenance of cellular integrity and mechanical homeostasis under the optimized culture conditions, while providing valuable insights into the 3D cellular architecture and intercellular organization within the spheroid model. Furthermore, SEM images provided enhanced visualization of the spatial relationship between CSMs and cell spheroids, while enabling detailed characterization of spheroid surface morphology and structural features, as demonstrated in Figure 5G–J. To further validate this relationship, a CSM containing cell spheroid was cut and observed under a light microscope. The captured images revealed that the spheroid was detached within the CSM, thereby confirming their spatial arrangement (Supporting Information S1: Figure S5).

**FIGURE 6:**
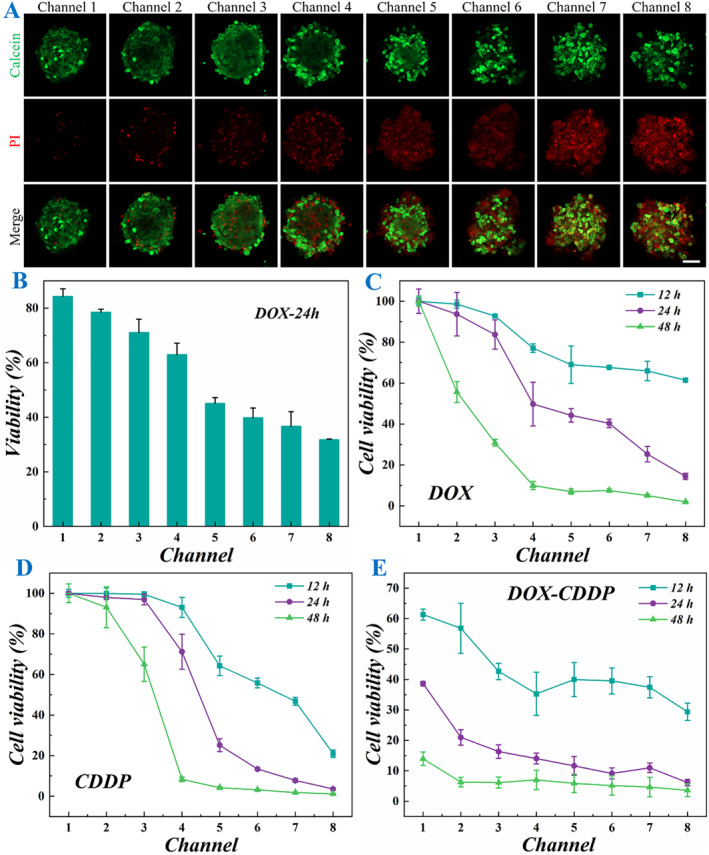
Results of OS‐on‐a‐chip for drug evaluation. (A) Live‐dead fluorescence images of cell spheroids in 8 channels after the chip was energized with DOX treatment for 24 h. (B) Quantification of live and dead cells in cell spheroids in 8 channels (*n* = 3 for each group). (C–E) The cell viability was quantified by CCK‐8 assay after 12, 24, and 48 h of DOX (C), CDDP (D), and DOX‐CDDP (E) treatment (*n* = 3 for each group). Scale bar is 50 μm in (A).

To systematically evaluate the drug sensitivity of the OS‐on‐a‐chip, we selected three clinically relevant chemotherapeutic agents, doxorubicin (DOX), cisplatin (CDDP), and ifosfamide (IFO), to establish a gradient model of bone tumor cell damage. Utilizing the microfluidic platform, we generated eight distinct drug concentration gradients within the same chip by introducing the selected drugs at one inlet and cell culture medium at the other inlet. This design ensured that all eight cell spheroids were initially identical but exposed to varying drug concentrations due to the diffusion gradient established within the chip. The drug‐induced cellular responses were quantitatively assessed through two validation methods, including live‐dead cell staining for morphological evaluation and CCK‐8 assay for cell viability measurement. The results demonstrate a clear dose‐dependent relationship between drug concentration and OS cell spheroid damage. As demonstrated in Figure 6A,B, Supporting Information S1: Figures S6 and S7, the damage extent of the cell spheroids progressively increased from channel 1 to channel 8 following the addition of DOX due to the gradient drug concentration. Furthermore, the cell viability assay revealed a consistent trend in spheroid viability reduction following DOX treatment (Figure 6B). Specifically, viability decreased significantly across channels 1–8 at 12, 24, and 48 h post‐treatment. Notably, the rate of viability reduction accelerated with prolonged exposure time, indicating the time‐dependent cytotoxic effects of DOX on OS spheroids (Figure 6C). Furthermore, to assess the broader applicability of our OS chip, we evaluated its generalizability by testing additional antitumor agents, namely CDDP and IFO (Figure 6D and Supporting Information S1: S8A). The results were consistent with that of the DOX group, demonstrating the successful development of the OS‐on‐a‐chip that could serve as a reliable platform for evaluating the efficacy of single antitumor drugs.

To evaluate the capability of the OS‐on‐a‐chip for assessing the combined effects of multiple antitumor drugs, we introduced cell culture media containing different drugs simultaneously at both inlets. This approach enabled the assessment of their potential synergistic or antagonistic interactions. As shown in Figure 6E, the DOX concentration decreased gradually from channel 1 to channel 8, while the CDDP concentration decreased progressively from channel 8 to channel 1. The results indicated that the potency of high DOX concentration was weaker compared to that of high CDDP concentration. However, CDDP typically exhibits strong organ toxicity, and thus, its clinical application can be optimized through the combined use of multiple drugs, which not only reduces the individual drug concentrations but also maintains or even enhances the overall therapeutic efficacy. This trend was consistently observed in other drug combination applications as demonstrated in Supporting Information S1: Figure S8B. Therefore, the developed 00‐on‐a‐chip can serve as an efficient platform for exploring and optimizing drug combinations, offering significant potential for clinical drug pairing strategies.

## 4 Conclusion

In this study, we present a novel approach utilizing microfluidic electrospray technology for the efficient preparation of CSMs loaded with OS cell spheroids. The combination of them and a multichannel microfluidic chip enabled the fabrication of a robust OS‐on‐a‐chip platform for high‐throughput drug screening and evaluation. The results demonstrated that the microfluidic electrospray technique facilitated the rapid and scalable production of homogeneous microcarriers, ensuring consistent and reproducible experimental conditions. Furthermore, the OS‐on‐a‐chip system was successfully validated for both single and multiple antitumor drug screening, showcasing its significant potential for clinical translation and therapeutic development. However, limitations such as the need for further validation with diverse drug types and patient‐derived models should be addressed in future work. Overall, this technology represents a promising tool for advancing precision medicine in OS research.

## Author Contributions

P. Lu, M. Zhao, and H. Wang conceived the idea. H. Su designed and conducted the experiments and data analysis. Y. Chen and H. Ren assisted the experiment and contributed to the scientific discussion. H. Su wrote the manuscript. Z. Xuan and H. Wang revised the manuscript.

## Ethics Statement

The authors have nothing to report.

## Conflicts of Interest

The authors declare no conflicts of interest.

## Supporting information

Supporting Information S1

## Data Availability

The data that support the findings of this study are available from the corresponding author upon reasonable request.
