# Supplementary material for: Permeable Hydrogel Encapsulated Osteosarcoma‐on‐a‐Chip for High‐Throughput Multi‐Drugs Screening
Source: Smart Med. 2025 Jul 12;4(3):e70013. doi: 10.1002/smmd.70013 (PMC12257891; doi:10.1002/smmd.70013)
Supplement: Supplementary file 1 — Supporting Information S1 [file SMMD-4-e70013-s001.docx]

Supporting Information

Permeable Hydrogel Encapsulated Osteosarcoma-On-A-Chip for High-Throughput Multi-Drugs Screening

*Haiwen Su, Yuanhai Chen, Zhiyan Xuan, Haoyu Ren, Peihua Lu^*^, Miaoqing Zhao^*^, Huan Wang^*^*


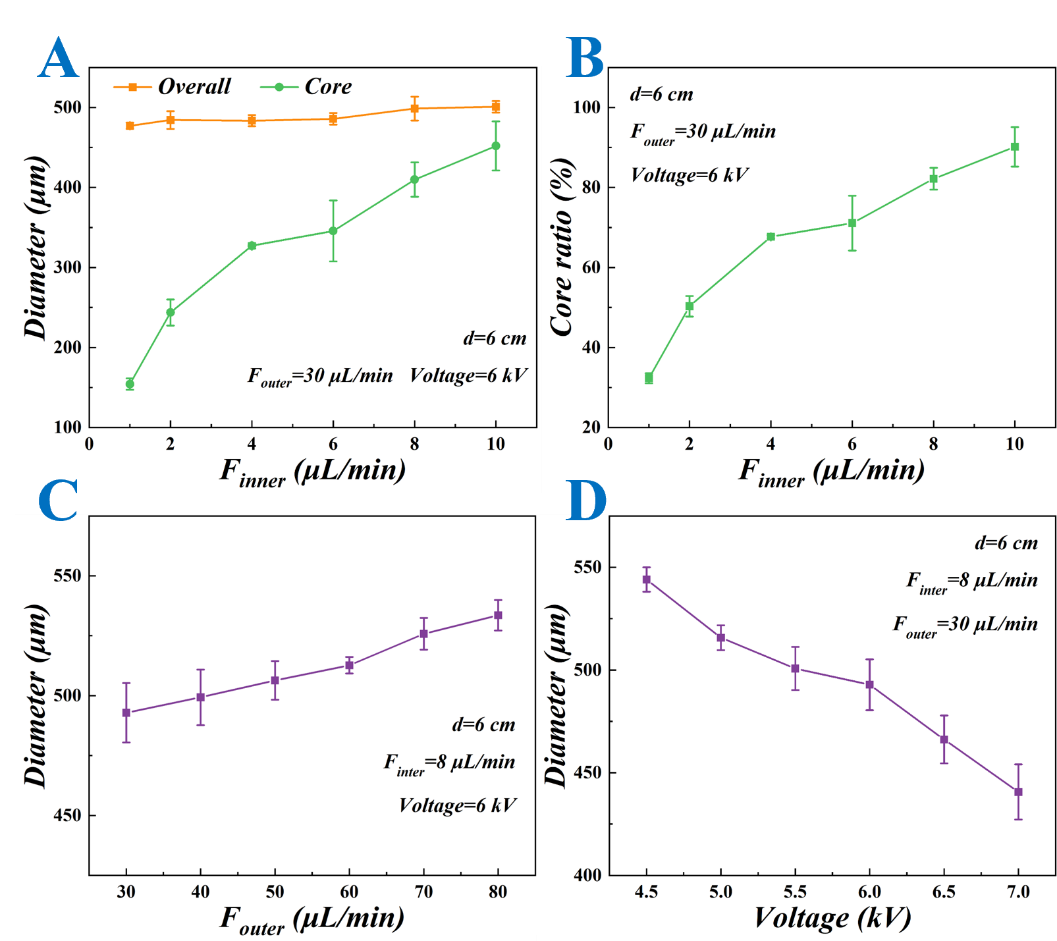


**Figure S1** Optimization of conditions for CSMs preparation. (A, B) The diameters (A) and core ratios (B) of CSMs prepared at different inner-phase flow rates. (C) The diameters of CSMs prepared at different outer-phase flow rates. (D) The diameter of CSMs prepared at different voltages.


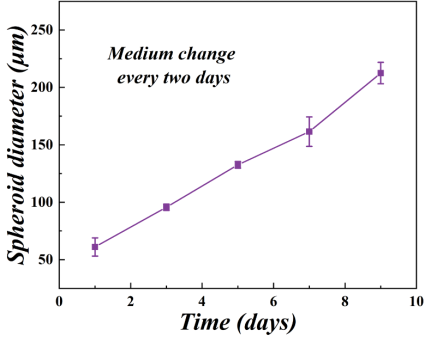


**Figure S2** Recorded increase in sphere diameter with days of growth.


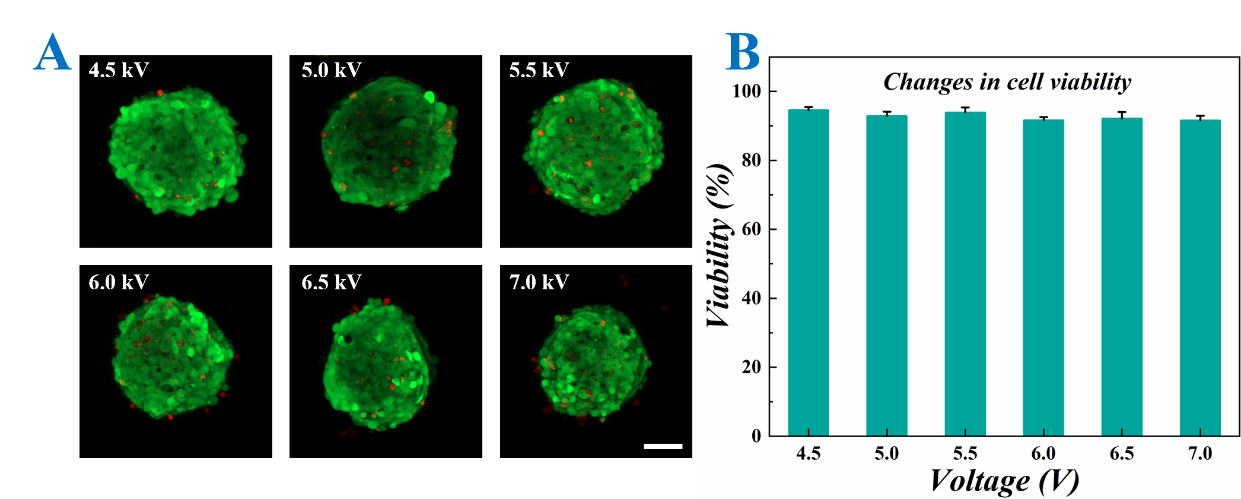


**Figure S3** Live and dead images (A) and quantitative analysis (B) of cell spheroids prepared at different voltages. Scale bar is 50 μm.


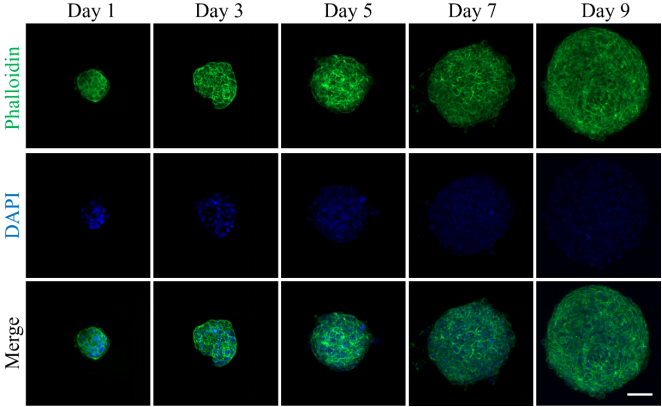


**Figure S4** Skeleton images of cell spheroids on days 1, 3, 5, 7, and 9. Scale bar is 50 μm.


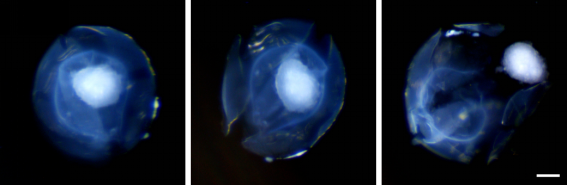


**Figure S5** Stereomicroscopic images of cell spheroids stripped from CSMs. Scale bar is 100 μm.


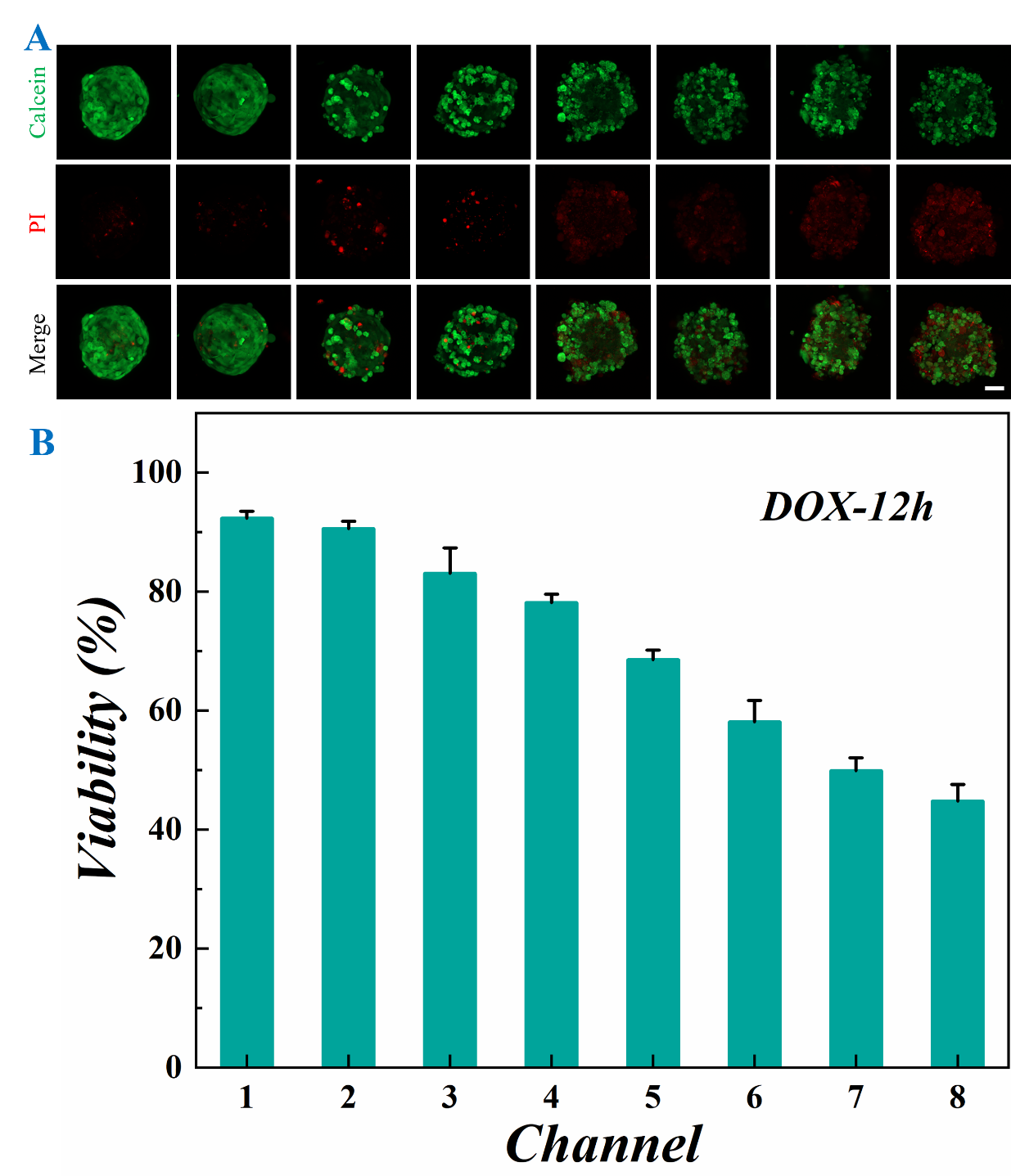


**Figure S6** Live-dead images (A) and quantitative analysis (B) of DOX treatment for 12h. Scale bar is 50 μm in (A).


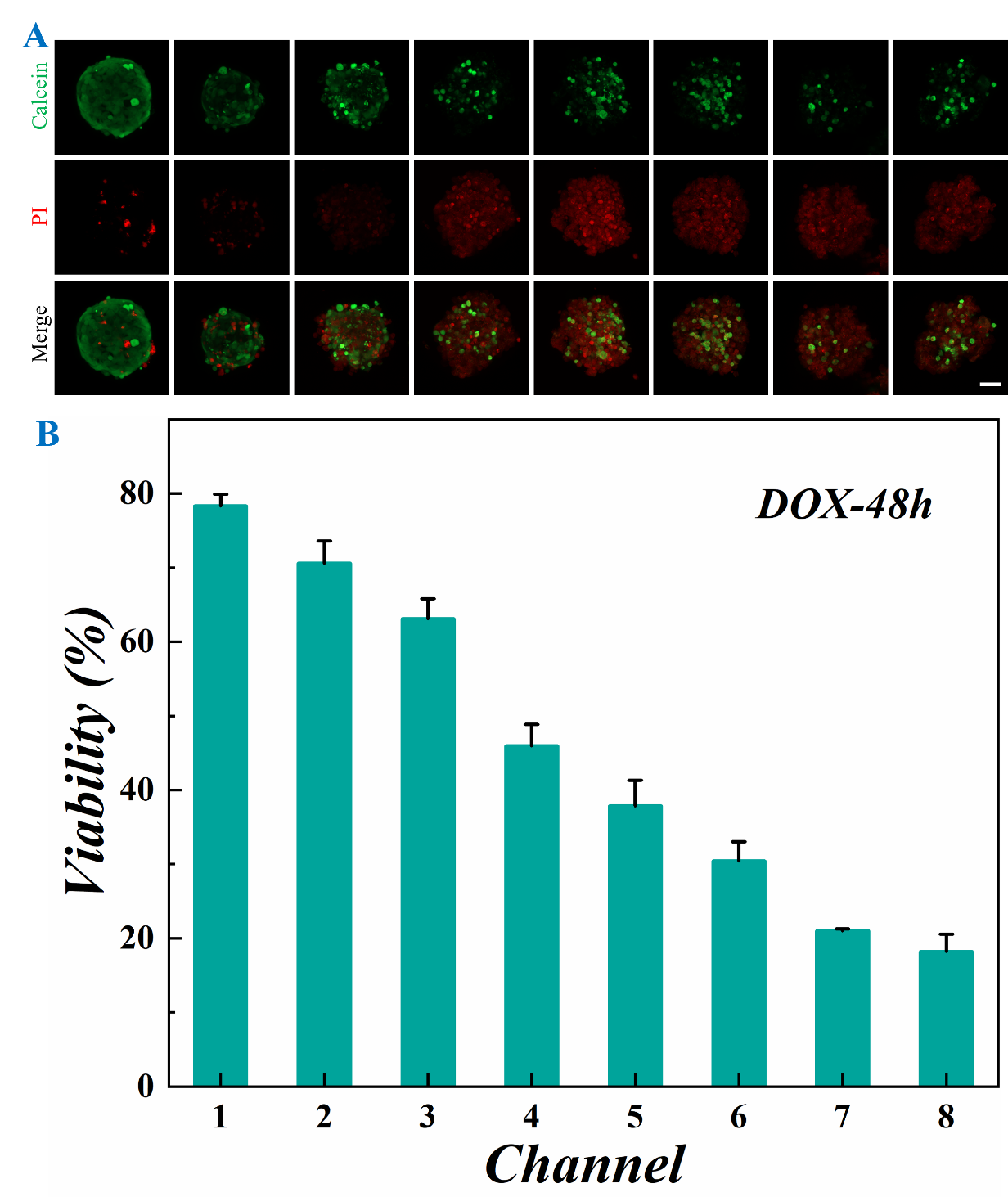


**Figure S7** Live-dead images (A) and quantitative analysis (B) of DOX treatment for 48h. Scale bar is 50 μm in (A).


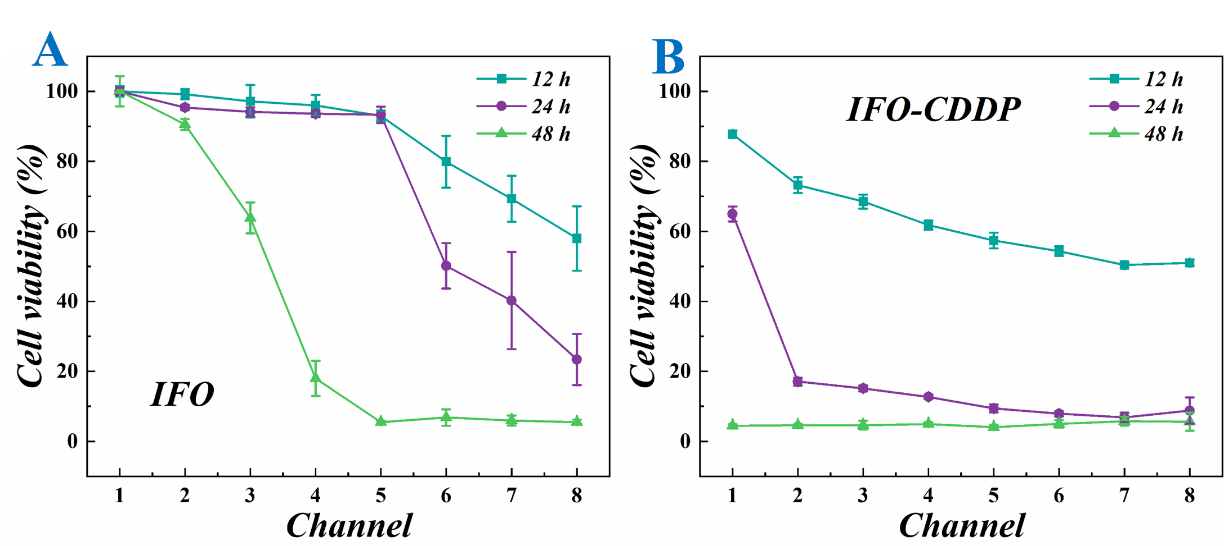


**Figure S8** The cell viability was quantified by CCK-8 assay after 12, 24, and 48 h of IFO (A) and IFO-CDDP (B) treatment.
